# Restoring the dampened expression of the core clock molecule BMAL1 protects against compression-induced intervertebral disc degeneration

**DOI:** 10.1038/s41413-022-00187-z

**Published:** 2022-02-25

**Authors:** Dong Wang, Pandi Peng, Michal Dudek, Xueyu Hu, Xiaolong Xu, Qiliang Shang, Di Wang, Haoruo Jia, Han Wang, Bo Gao, Chao Zheng, Jianxin Mao, Chu Gao, Xin He, Pengzhen Cheng, Huanbo Wang, Jianmin Zheng, Judith A. Hoyland, Qing-Jun Meng, Zhuojing Luo, Liu Yang

**Affiliations:** 1 Institute of Orthopedic Surgery, Xijing Hospital, Fourth Military Medical University, Xi’an, 710032 People’s Republic of China; 2 Medical Research Institute, Northwestern Polytechnical University, Xi’an, 710068 People’s Republic of China; 3 School of Biological Sciences, Faculty of Biology, Medicine and Health, University of Manchester, Manchester, M13 9PL UK; 4 Wellcome Centre for Cell Matrix Research, University of Manchester, Manchester, M13 9PL UK; 5 Department of Medicine Chemistry and Pharmaceutical Analysis, School of Pharmacy, Fourth Military Medical University, Xi’an, 710032 People’s Republic of China; 6 Radiology Department, Xijing Hospital, Fourth Military Medical University, Xi’an, 710032 People’s Republic of China

**Keywords:** Pathogenesis, Diseases

## Abstract

The circadian clock participates in maintaining homeostasis in peripheral tissues, including intervertebral discs (IVDs). Abnormal mechanical loading is a known risk factor for intervertebral disc degeneration (IDD). Based on the rhythmic daily loading pattern of rest and activity, we hypothesized that abnormal mechanical loading could dampen the IVD clock, contributing to IDD. Here, we investigated the effects of abnormal loading on the IVD clock and aimed to inhibit compression-induced IDD by targeting the core clock molecule brain and muscle Arnt-like protein-1 (BMAL1). In this study, we showed that BMAL1 KO mice exhibit radiographic features similar to those of human IDD and that BMAL1 expression was negatively correlated with IDD severity by systematic analysis based on 149 human IVD samples. The intrinsic circadian clock in the IVD was dampened by excessive loading, and BMAL1 overexpression by lentivirus attenuated compression-induced IDD. Inhibition of the RhoA/ROCK pathway by Y-27632 or melatonin attenuated the compression-induced decrease in BMAL1 expression. Finally, the two drugs partially restored BMAL1 expression and alleviated IDD in a diurnal compression model. Our results first show that excessive loading dampens the circadian clock of nucleus pulposus tissues via the RhoA/ROCK pathway, the inhibition of which potentially protects against compression-induced IDD by preserving BMAL1 expression. These findings underline the importance of the circadian clock for IVD homeostasis and provide a potentially effective therapeutic strategy for IDD.

## Introduction

The circadian clock is an evolutionarily conserved internal timekeeping system that maintains body physiology and behavior in a constant 24 h diurnal cycle.^1^ The central pacemaker in suprachiasmatic nuclei (SCN) is entrained by external environmental cues (also called zeitgebers) and synchronizes peripheral clocks in almost all tissues and organs.^1–3^ Subsequently, circadian clocks in peripheral tissues maintain tissue homeostasis by rhythmically regulating different clock-controlled genes in a tissue-specific manner.^4–6^ Cell-autonomous molecular circadian clocks are complex and contain interlocking transcription–translation feedback loops (TTFLs). *Bmal1* is an essential core clock gene in TTFLs, the knockout of which disrupts rhythmic oscillation and abolishes the CLOCK-BMAL1 heterodimer’s transcriptional function.^1,7–9^ Circadian clock disruption is reportedly closely associated with many diseases, such as cancer, cardiovascular diseases, neurodegenerative disorders, liver and kidney dysfunction, and osteoarthritis.^10–14^ However, the role of the circadian clock in the development of intervertebral disc degeneration (IDD) is still unclear.

Low back pain (LBP) and IDD are extremely common symptoms in people of all ages, affecting approximately 568 million people worldwide.^15,16^ Due to the high prevalence and years of life lived with disability associated with musculoskeletal diseases, LBP has imposed a considerable disease burden in most countries.^16,17^ As an axial bone, the spine provides mechanical support to the entire body. Mechanical loading also plays an important role in spine development and maintenance of spinal homeostasis.^18–20^ Current evidence suggests that accumulated mechanical stresses, especially excessive compressive stress, impair extracellular matrix (ECM) metabolism and induce nucleus pulposus (NP) cell apoptosis, thus contributing to ECM degradation and ultimately leading to IDD.^21–25^ However, the mechanism underlying IDD induced by abnormal mechanical loading has not been fully elucidated.

The IVD is a highly rhythmic tissue that experiences a diurnal activity/rest cycle.^26,27^ Shift work, which disrupts the diurnal rhythmic cycle, is considered a risk factor for LBP.^28,29^ Previously, our group showed that IVDs contain an intrinsic circadian clock, which could be regulated by aging and disrupted by IL-1β.^30^ However, the effect of abnormal mechanical loading on the IVD circadian clock and the role it plays in IDD development and progression have not been explored.

In this study, we systemically analyzed the relationship between BMAL1 expression and IDD severity in human NP tissue and further demonstrated the participation of the dampened peripheral clock in IDD development induced by excessive mechanical loading. Moreover, we showed that excessive loading activated the RhoA/ROCK pathway, thus leading to decreased BMAL1 expression in NP cells. RhoA/ROCK pathway inhibition by Y-27632 and melatonin partially rescued BMAL1 expression and successfully ameliorated IDD progression.

## Results

### BMAL1 expression is negatively correlated with IDD severity

**Fig. 1:**
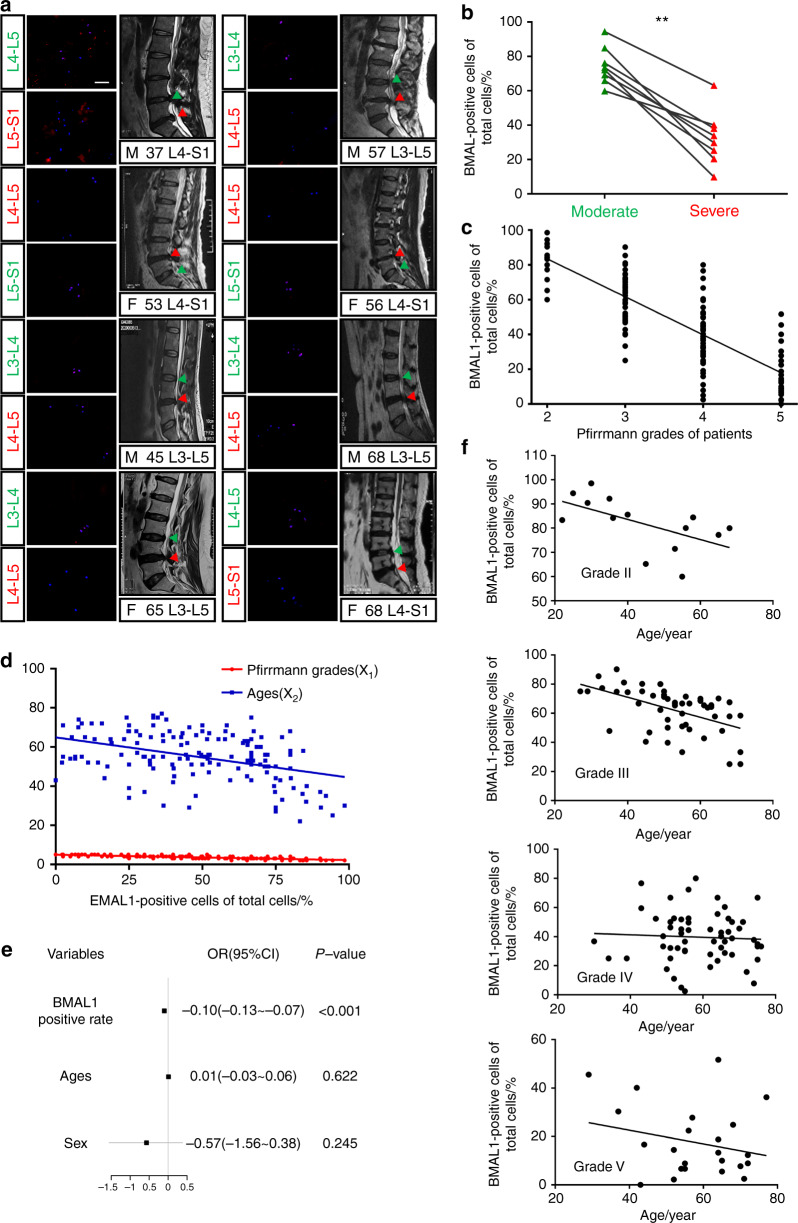
BMAL1 shows a negative correlation with the degree of IDD. **a** Immunofluorescence of BMAL1 in 8 pairs of human NP samples with different IDD severities from each patient. Scale bar: 50 μm. Green triangles: IVDs with moderate IDD severity; red triangles: IVDs with severe IDD severity. *n* = 8. **b** Quantification of the percentage of BMAL1-positive cells in 8 pairs of human NP samples with different IDD severities from each patient. Paired Student’s *t* test. **c** Single-factor linear regression analysis of the relationship between the percentage of BMAL1-positive cells and Pfirrmann grades based on 149 NP samples. **d** Multivariate linear regression analysis of the relationship between all variables (Pfirrmann grades and ages) and the percentage of BMAL1-positive cells. **e** Logistic regression analyses for the relationship between all variables (BMAL1 positive rate, sex and age) and IDD severity. **f** Single-factor linear regression analysis of the relationship between the percentage of BMAL1-positive cells and age for NP tissues of similar Pfirrmann grades. **P < 0.01

In our previous study, we showed that human NP cells express BMAL1 and CLOCK proteins.^30^ However, the relationship between BMAL1 expression in human NP tissue and IDD severity remains unclear. Therefore, we collected 149 NP specimens with different Pfirrmann grades to detect BMAL1 expression (all information for the patients is shown in Table S1). To avoid the effect of interpatient variability, we performed a comparison between pairs of discs with different IDD severities from each patient (Grade II and III samples were combined into a moderate group, while Grade IV and V samples were combined into a severe group). All eight patients had a moderate IDD segment and a severe segment. Human NP tissue immunofluorescence staining and MRI from the eight patients revealed that BMAL1 expression in the severely degenerative segments was significantly lower than that in the moderate group (Fig. 1a, b). Single-factor linear regression for the relationship between the percentage of BMAL1-positive cells and Pfirrmann grades in all 149 NP samples revealed a negative correlation between BMAL1 expression and IDD Pfirrmann grades (y = –21.86x + 127.2, *r* = 0.605 9, *P* < 0.000 1; Fig. 1c).

Age and sex are reportedly two factors that affect IDD occurrence and development^31,32^. To examine the influence of age and sex on BMAL1 expression, we performed Multivariate linear regression analysis. Age and Pfirrmann grades were the two main negatively correlated factors for BMAL1 expression, and sex was an irrelevant factor (y = 140.854 –0.722×_1_–0.178×_2_, Pfirrmann grade (x_1_), age (x_2_), *P* < 0.000 1; Fig. 1d).

Considering the negative correlation of IDD with BMAL1 expression, we explored whether BMAL1 expression was an important predictor of IDD progression. Logistic regression analysis demonstrated that BMAL1 expression was a protective factor against IVD homeostasis (Fig. 1e and Table S2). However, age did not contribute to IDD development, which seemed contradictory to its contribution to BMAL1 expression. Therefore, we analyzed the relationship between BMAL1 expression and age by single-factor linear regression analysis for NP tissues with a similar Pfirrmann grade (Fig. 1f). Age was negatively correlated with BMAL1 expression only in the moderate IDD group (Grade II: *y* = –0.414 4x + 100.2, *r* = 0.334 4, *P* = 0.030 3; Grade III: *y* = –0.688 2x + 98.52, *r* = 0.266, *P* < 0.000 1; Grade IV: *y* = –0.085 25x + 44.71, *r* = 0.002 829, *P* = 0.689 1; Grade V: *y* = –0.283 3x + 33.93, *r* = 0.058 47, *P* = 0.255 0), suggesting that age as a factor influences the expression of BMAL1 in the early stage of IDD, while other factors, such as abnormal loading, may play a more significant role in the later progression of IDD.

### Excessive mechanical loading disrupts the IVD circadian clock

**Fig. 2:**
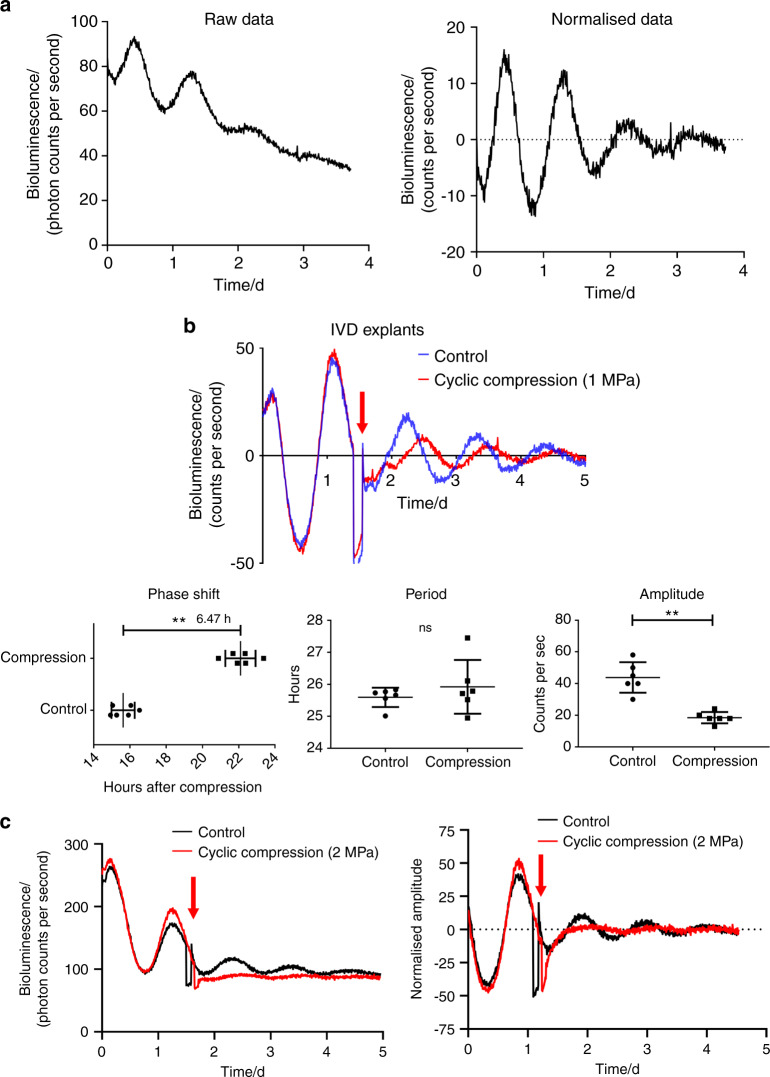
Excessive mechanical loading dampens the circadian rhythm of IVDs. **a** Representative PER2::Luc bioluminescence trace of human primary NP cells transduced with lentiviral *Per2*::Luc. *n* = 6. **b** Representative PER2::Luc bioluminescence trace of mouse IVD explant culture with cyclic compression (1.0 MPa, 1.0 Hz, 2 h) and quantification of phase, period and amplitude. The red arrow at Day 1.5 indicates the time of mechanical loading. *n* = 6. **c** Representative PER2::Luc bioluminescence trace of mouse IVD explant culture with cyclic compression (2.0 MPa, 1.0 Hz, 1 h). The red arrow at Day 1.5 indicates the time of mechanical loading. *n* = 6. ns not significant, **P < 0.01

To confirm that human NP tissue has a functioning circadian clock, we transduced human primary NP cells with lentiviral *Per-Luc* and monitored their bioluminescence for 4 days (Fig. 2a). Indeed, autonomous rhythmic oscillations were observed in the NP tissue. To test whether excessive mechanical loading dampens IVD circadian rhythm, we subjected IVD explants isolated from *PER2::Luc* reporter mice to a cyclic compression protocol (1.0 MPa, 1 Hz, and 2 h). Compared with the noncompressed IVD explants, the compressed explants demonstrated a large reduction in circadian amplitude and significant phase delay but no change in period (Fig. 2b). Moreover, a further increase in the magnitude of mechanical loading (2.0 MPa, 1 Hz, and 1 h) resulted in a complete loss of circadian rhythm in mouse IVD explants (Fig. 2c).

### Overexpression of BMAL1 partially prevents compression-induced NP cell apoptosis and dysfunction

**Fig. 3:**
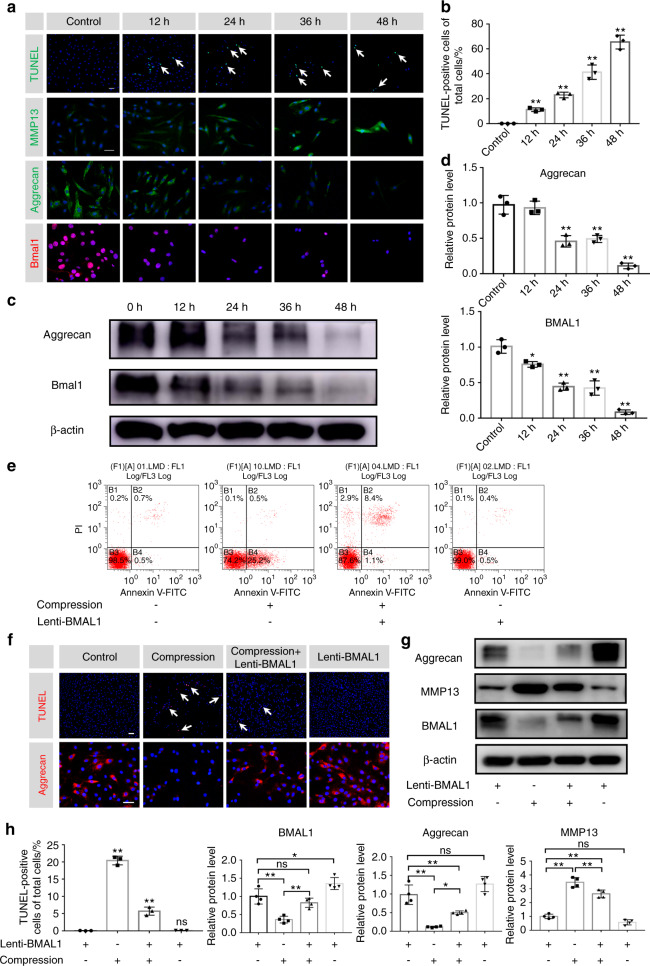
Overexpression of BMAL1 prevents compression-induced NP cell apoptosis and dysfunction. NP cells were treated with 1.0 MPa compression for 0, 12, 24, 36 and 48 h, and some results are shown in the following graphs. **a** The expression of BMAL1, Aggrecan and MMP13 detected by immunofluorescence and apoptosis detected by TUNEL staining. Scale bar: 50 μm. White arrows: TUNEL-positive cells. **b** Quantification of the percentage of TUNEL-positive cells. **c, d** The protein expression of Aggrecan and BMAL1 was detected by western blots in NP cells. The following graphs based on the experiments in which NP cells were subjected to Lent-BMAL1 transfection and then to 1.0 MPa compression for 0 and 24 h. **e** Representative dot plot of cell apoptosis by flow cytometry analysis after Annexin V/PI dual staining. *n* = 3. **f** The expression of Aggrecan detected by immunofluorescence and the apoptosis detected by TUNEL staining. Scale bar: 50 μm. *n* = 3. White arrows: TUNEL-positive cells. **g** The protein expression of Aggrecan, MMP13 and BMAL1 was detected by western blot in NP cells. *n* = 4. **h** Quantification of the percentage of TUNEL-positive cells and the protein expression of Aggrecan, MMP13 and BMAL1. The data in the figures represent the mean ± S.D. ns not significant, **P < 0.01, *P < 0.05, *n* = 3. Differences among multiple groups were analyzed by one-way ANOVA

Based on the negative correlation of IDD with BMAL1 expression and the ability of excessive compression to disrupt the IVD circadian rhythm, we hypothesized that recovery of dampened BMAL1 expression may partially ameliorate excessive compression-induced dysfunction of NP cells. To test the detrimental effect of excessive compression on BMAL1 expression and ECM homeostasis, we placed NP cells in a compression culture chamber. The cells underwent 1.0 MPa static compression for 0, 12, 24, 36, and 48 h. Immunofluorescence staining revealed a gradual decrease in BMAL1 expression over time. IVD homeostasis was also disturbed, which was confirmed by the imbalance between anabolism and catabolism (decreased aggrecan expression and increased MMP13 expression) and increased NP cell apoptosis (Fig. 3a, b and Fig. S1a). Western blot analysis also revealed reduced BMAL1 and aggrecan expression (Fig. 3c, d). These results indicate that excessive compression impairs BMAL1 expression and leads to dysfunction of NP cells.

To investigate whether increasing *Bmal1* expression could rescue compression-induced NP cell dysfunction, we infected NP cells with Lenti-BMAL1 to overexpress BMAL1. Annexin-V/PI and terminal deoxynucleotidyl transferase dUTP nick end labeling (TUNEL) staining assays showed that BMAL1 overexpression attenuated compression-induced NP cell apoptosis. Moreover, immunofluorescence staining showed partially rescued aggrecan expression in the compression + Lenti-BMAL1 group (Fig. 3e, f and Fig. S1b). Western blot assays also confirmed that BMAL1 overexpression restored NP cell metabolic homeostasis under compression stress (Fig. 3g, h). These results suggest that loss of BMAL1 expression and disruption of the circadian clock are important contributors to compression-induced NP cell dysfunction. Upregulating BMAL1 expression is an effective method to prevent compression-induced NP cell apoptosis and dysfunction.

### *Bmal1* deletion in mice leads to NP degeneration

**Fig. 4:**
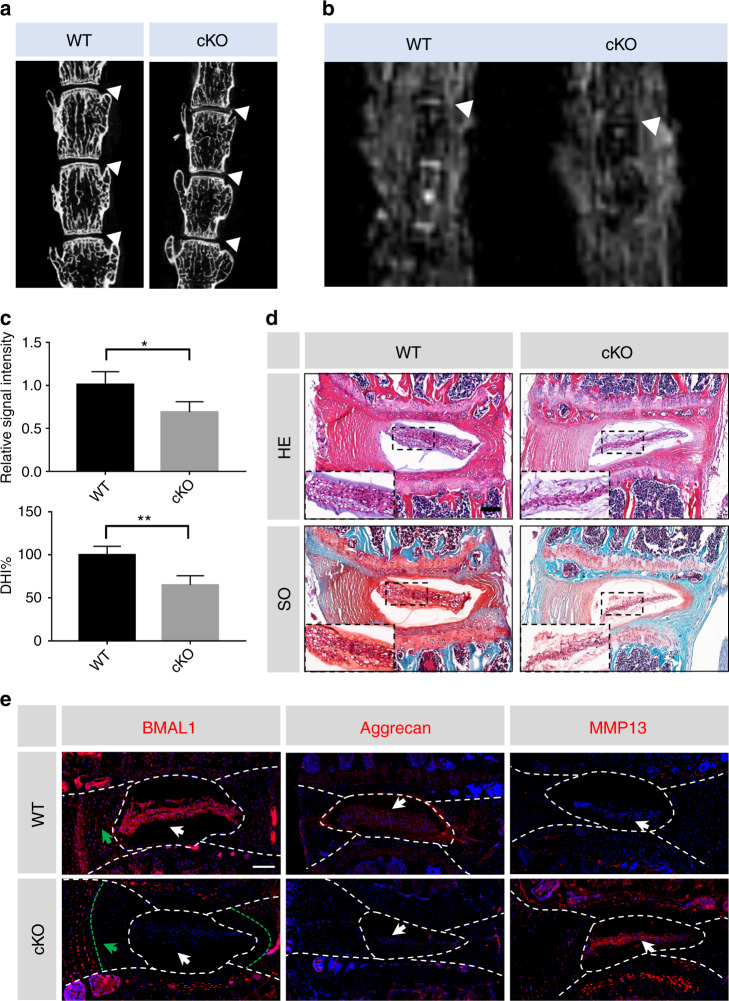
Deletion of *Bmal1* leads to the degradation of ECM in NP tissue. **a** Representative µCT scans of the lumbar spine of a WT mouse and a KO littermate (6 months). White triangles: lumbar IVDs. *n* = 4. **b** Representative MRI scan of the mouse lumbar spine. White triangles: lumbar IVDs. *n* = 3. **c** Quantification of the relative signal intensity of MRI and DHI% of µCT. Student’s *t* test. **d** HE and SO staining of the IVDs from the lumbar spine of a WT mouse and a KO littermate (6 months). Scale bar: 100 μm. **e** Representative images of immunofluorescence of BMAL1, Aggrecan and MMP13. White arrows: NP tissues; green arrows: inner AF tissues. Scale bar: 100 μm. The data in the figures represent the mean ± S.D. **P < 0.01, *P < 0.05

In our previous study, we characterized the phenotype of IVD-specific BMAL1 KO mice (*Col2a1*^*Cre*^*Bmal1*^*fl/fl*^, cKO) and described degeneration of the outer annulus fibrosus (AF).^30^ However, the effect of BMAL1 KO on NP tissue was not investigated. The cKO mice and their wild-type (WT) littermates were sacrificed at 6 months of age, and IVDs were used for subsequent analysis. We analyzed the lumbar disc height of the cKO and WT mice using microcomputed tomography. A markedly reduced disc height index (DHI, %DHI = cKO DHI/WT DHI × 100%) was observed in the cKO IVDs (Fig. 4a, c). To evaluate NP tissue water content (a major characteristic of normal ECM homeostasis in IVDs), we performed MRI in the WT and cKO mice. The T2-weighted images revealed a lower signal intensity in the cKO IVDs, implying a degraded ECM in the cKO NP tissues (Fig. 4b, c). Histological analysis using HE and SO staining also confirmed degenerative changes in the cKO NP tissues. Compared to the WT mice, the cKO mice showed enhanced proteoglycan loss in NP and inner AF tissues (Fig. 4d). The area filled with less proteoglycan in the cKO mouse IVDs was consistent with that filled with BMAL1-null cells (Fig. 4e). Immunofluorescence staining further revealed IDD molecular mechanisms induced by *Bmal1* deletion. Reduced aggrecan and increased MMP13 expression were observed in the cKO mice, indicating that *Bmal1* deficiency could result in ECM degradation, thus leading to IDD development (Fig. 4e). Therefore, BMAL1 is essential for NP homeostasis, the loss of which could induce IDD development.

### Upregulation of BMAL1 expression by inhibiting the RhoA/ROCK pathway ameliorates compression-induced apoptosis of NP cells and degradation of ECM in NP tissues

**Fig. 5:**
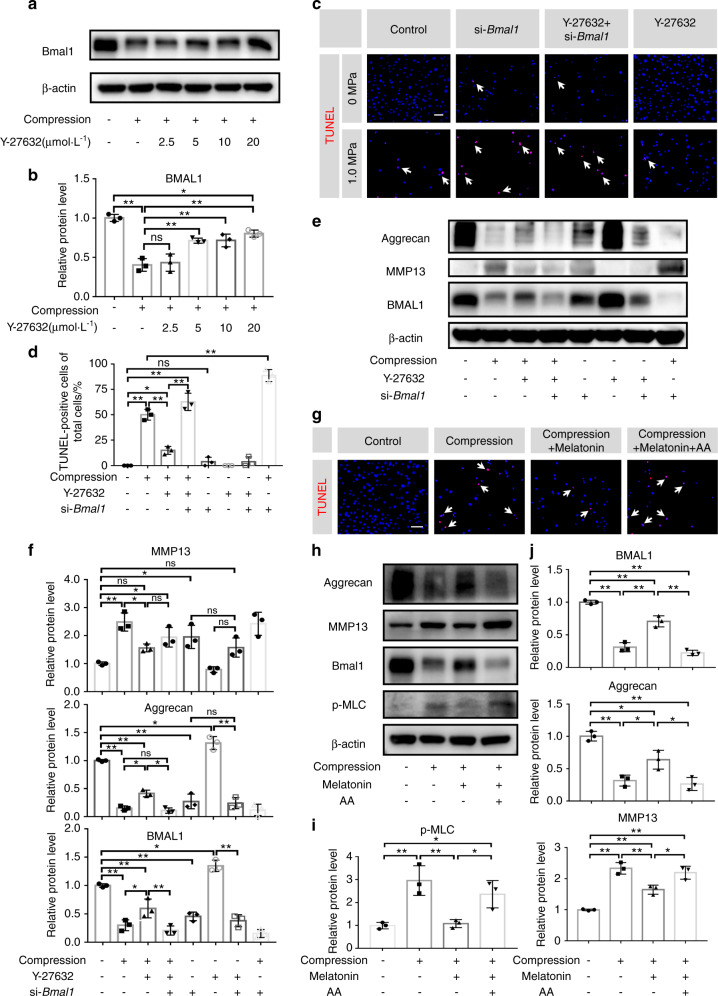
Upregulation of BMAL1 expression by inhibiting the RhoA/ROCK pathway ameliorates compression-induced apoptosis of NP cells and degradation of ECM in NP tissues. **a, b** The protein levels of BMAL1 in NP cells treated with compression and different doses of Y-27632. **c, d** The apoptosis level of NP cells detected by TUNEL staining. Scale bar: 50 μm. White arrows: TUNEL-positive cells. **e, f** The protein expression of Aggrecan, MMP13, and BMAL1 detected by western blots in NP cells. **g** The apoptosis level of NP cells detected by TUNEL staining. Scale bar: 50 μm. White arrows: TUNEL-positive cells. **h–j** The protein expression of Aggrecan, MMP13, BMAL1, and p-MLC detected by western blots in NP cells. The data in the figures represent the mean ± S.D. ns not significant, **P < 0.01, *P < 0.05, *n* = 3. Differences among multiple groups were analyzed by one-way ANOVA

The RhoA/ROCK pathway plays a pivotal role in mechanotransduction.^33,34^ Previous studies have shown that the activation of RhoA/ROCK signaling participates in cytoskeletal remodeling in chondrocytes and induces articular cartilage degradation.^35,36^ Hence, we hypothesized that RhoA/ROCK signaling may participate in mediating the effect of compression stress on the IVD circadian clock. To test our hypothesis, we used Y-27632 to inhibit the RhoA/ROCK pathway under compressive stress. Western blot assays showed that BMAL1 expression markedly decreased under compression stress and was gradually restored after Y-27632 treatment in a dose-dependent manner (Fig. 5a, b). To test whether Y-27632 rescued NP cell apoptosis and ECM degradation by upregulating BMAL1 expression, we transfected NP cells with BMAL1-siRNA before they received compression and Y-27632. A reduced TUNEL positivity rate was observed after Y-27632 treatment compared with that of the NP cells subjected to compression stress only. Moreover, the western blot assay revealed that Y-27632 partially rescued the compression-induced BMAL1 and aggrecan reduction and MMP13 increase in NP cells. si-*Bmal1* alone significantly reduced aggrecan expression and increased MMP13 expression. More importantly, the therapeutic effect of Y-27632 was completely abolished by BMAL1-siRNA transfection. Y-27632 supplementation did not attenuate the BMAL1 knockdown-induced disturbed metabolism (Fig. 5c–f). These results indicated that compression stress decreases BMAL1 expression through RhoA/ROCK pathway activation, and RhoA/ROCK pathway inhibition by Y-27632 partially attenuated the decreased expression of BMAL1 and thus ameliorated the process of IDD induced by compression stress.

Melatonin, a hormone secreted by the pineal gland, potentially restores the central circadian rhythm.^37^ However, the mechanism by which melatonin regulates peripheral clocks has not yet been elucidated. A previous study reported that melatonin could inhibit RhoA/ROCK pathway activation in choroidal neovascularization.^38^ To test whether melatonin restored BMAL1 expression and attenuated IDD development through RhoA/ROCK pathway inhibition, we used arachidonic acid (AA), a ROCK agonist that interacts with the ROCK regulatory region, in our experiments. Compared to that of the compression group, a reduced number of TUNEL-positive cells was observed after adding melatonin, and this effect was completely abolished by AA treatment (Fig. 5g). Additionally, we observed predominantly increased p-MLC expression (a RhoA/ROCK pathway activation marker) in the compression group using western blot assays. Melatonin reduced p-MLC expression, but this effect was blocked by AA (Fig. 5h, i). A 24-h 1.0 MPa compression resulted in decreased BMAL1 and aggrecan expression and increased MMP13 expression. Melatonin treatment prevented the BMAL1 reduction as well as changes in the expression of aggrecan and MMP13. This protective effect disappeared after AA addition (Fig. 5h, j). These results indicated that melatonin could prevent the downregulation of BMAL1 expression by inhibiting the RhoA/ROCK pathway.

Thereafter, we evaluated this mechanism using an organotypic tissue-explant culture. After a 6-day 1.0 MPa compression, no apparent histopathological change was observed in the compression group. However, an increased number of TUNEL-positive cells was observed using TUNEL staining. Both Y-27632 and melatonin partially reversed these phenotypes (Fig. S2a). After 12 days of compression treatment, obvious histological changes were observed in the compression group. An increased number of TUNEL-positive cells was also observed. Accordingly, both Y-27632 and melatonin also partially reversed these phenotypes (Fig. S2b). These results confirm that blocking the RhoA/ROCK pathway ameliorates compression-induced NP cell apoptosis and ECM degradation in NP tissues.

### Y-27632 and melatonin restored the expression of BMAL1 and ameliorated the IDD process in a compression-induced rat model

**Fig. 6:**
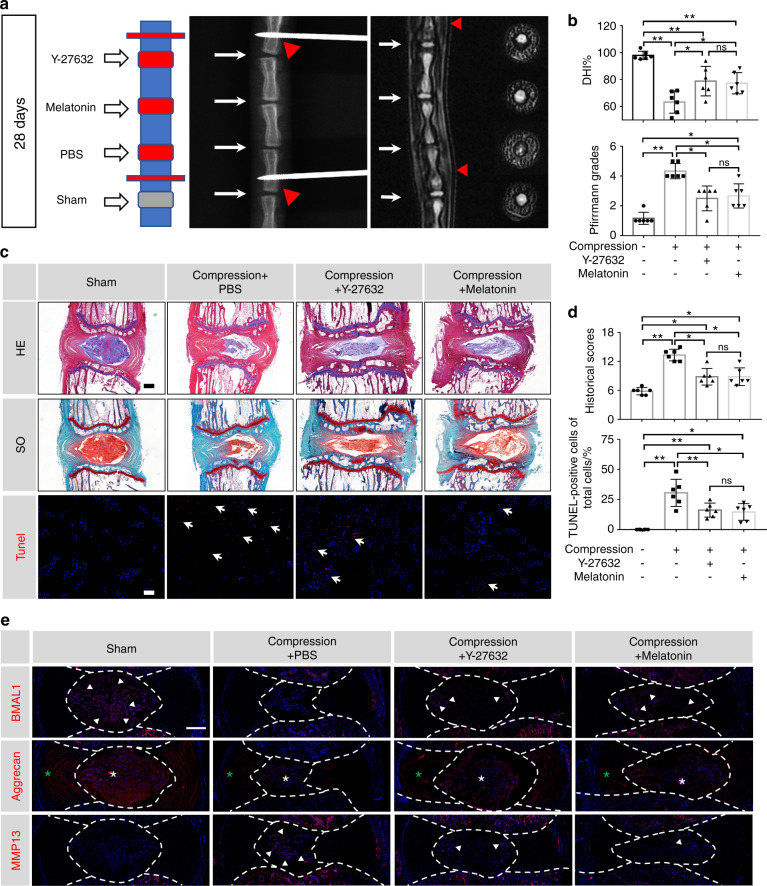
Y-27632 and melatonin restore the expression of BMAL1 and ameliorate the IDD process in a compression-induced rat model. **a** Representative X-ray and MRI images of rat coccygeal vertebrae on Day 28 after surgery and a schematic diagram of the operation. White arrows: points of injection; red sticks and red triangles: external fixation points; red squares: compressed discs; gray squares: uncompressed discs. **b** Statistical graphs of DHI% and Pfirrmann grades. Differences among multiple groups were analyzed by one-way ANOVA and Kruskal-Wallis h-tests. **c** Representative HE, SO, and TUNEL staining images. Scale bar: 500 μm. **d** Statistical graphs of histological scores and the percentage of TUNEL-positive cells. Differences among multiple groups were analyzed by Kruskal-Wallis h-tests and one-way ANOVA. **e** Representative images of immunofluorescence of BMAL1, Aggrecan and MMP13. Central parts of these graphs encompassed by white dotted lines indicate NP tissues. White arrows: BMAL1- or MMP13-positive cells. White asterisk: Aggrecan-positive area in NP tissue. Green asterisk: Aggrecan-positive area in AF tissue. The data in the figures represent the mean ± S.D. ns not significant, **P < 0.01, *P < 0.05, *n* = 6

To investigate the effects of Y-27632 and melatonin on IDD in vivo, we established a novel compression-induced rat model, followed by intradiscal administration of 20 μmol·L^−1^ Y-27632 or 500 μmol·L^−1^ melatonin semiweekly for 4 weeks. Radiography revealed a significantly decreased DHI in the compression + PBS group compared to that in the sham group on Day 28. Partially recovered DHIs in the compression + Y-27632 and compression + melatonin groups were observed on Day 28. MRI also revealed evidently higher Pfirrmann grade scores in the compression + PBS group; however, treatment with Y-27632 or melatonin significantly decreased these scores after 28 days (Fig. 6a, b).

Additionally, HE and SO staining revealed a reduction in NP cell number in the compression + PBS group after 28 days of compression, accompanied by disorganized AF lamellae and collapsed CEPs, while both Y-27632 and melatonin treatments ameliorated histological IDD changes. Moreover, TUNEL staining revealed an increase in TUNEL-positive cells in NP tissue in the compression + PBS group, which was attenuated by either Y-27632 or melatonin treatment (Fig. 6c, d). To detect the protective effect of Y-27632 and melatonin on ECM and BMAL1 expression, we performed immunofluorescence staining. BMAL1 and aggrecan expression was significantly reduced in NP tissues after 28 days of compression, accompanied by increased MMP13 expression, whereas both Y-27632 and melatonin treatments partially attenuated ECM dyshomeostasis and recovered BMAL1 expression in NP tissues (Fig. 6e and Fig. S3). These results further demonstrate the therapeutic effects of Y-27632 and melatonin in vivo.

## Discussion

**Fig. 7:**
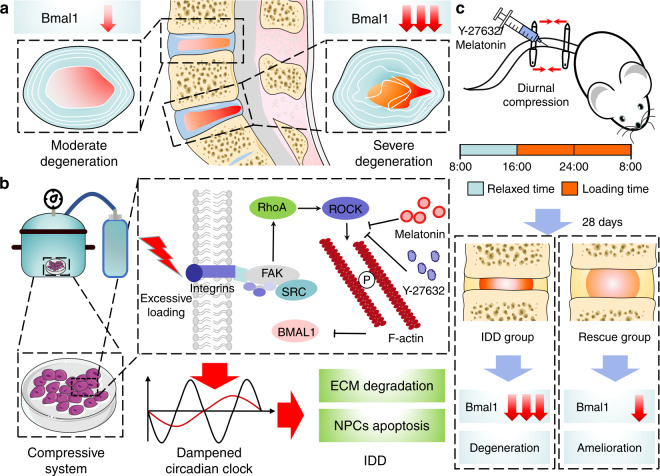
Proposed schematic representation for this study. **a** Clinical sample collection and analysis of BMAL1 levels. **b** Molecular mechanism of excessive loading dampening the circadian clock of IVDs and upregulating BMAL1 expression by RhoA/ROCK pathway inhibition to attenuate compression-induced IDD. **c** Melatonin and Y-27632 partially restored BMAL1 expression and alleviated IDD in a diurnal compression model

The mammalian circadian clock is a coordinated network of hierarchical oscillators. The central clock can relay timing cues to peripheral tissues and affect their circadian oscillations through neurohumoral regulation.^3,39^ Our study innovatively selected eight sample pairs with different IDD severities in the same individual, which successfully eliminated the differences caused by the central clock and many other individual differences (Fig. 7a). Previous studies have shown that core clock proteins exist in some human peripheral tissues, and reduced BMAL1 expression was observed in human knees with osteoarthritis. However, correlation studies between BMAL1 expression and IDD are still lacking.

The circadian clock can reportedly be dampened with aging.^40,41^ In our study, we confirmed that BMAL1 expression was negatively correlated with age. This result was consistent with our previous work demonstrating that aging dampened IVD circadian oscillation in mice.^30^ However, logistic regression analysis revealed that age was not involved in IDD progression. These results suggest that in addition to age, local abnormal factors (e.g., excessive mechanical loading) may also participate in BMAL1 expression reduction and IDD progression. Our cKO model also showed that local BMAL1 loss induced ECM degradation in NP tissues. These results support our hypothesis that decreased BMAL1 expression contributes to IDD progression.

Circadian clock disruption has been proven to increase the risk of many diseases, including obesity, cardiovascular diseases, and osteoarthritis.^4,42–45^ Human NP sample results revealed a negative correlation between BMAL1 expression and IDD severity. However, confirming the disrupted IVD circadian clock as the cause of IDD or the result of compression-induced IDD is challenging. In our study, BMAL1 deletion in NP tissues by cKO mice resulted in ECM degradation in NP tissue and disc height loss, further confirming the disrupted circadian clock as a mediator between excessive loading and IDD. Therefore, our study is the first to confirm that excessive mechanical loading dampens the IVD circadian rhythm and inhibits BMAL1 expression, finally leading to disc degeneration (Fig. 7b).

Moreover, we successfully revealed that the RhoA/ROCK pathway mediated decreased BMAL1 expression caused by excessive mechanical loading. Previously, our group demonstrated that tissue stiffness potentially controled circadian clocks, and RhoA/ROCK pathway inhibition could enhance the transactivation of reporters containing the E-box sequence in mammary cells.^46^ In this study, our results demonstrated that 24-h 1.0 MPa compression potentially activated the RhoA/ROCK pathway in NP cells, and pathway inhibition by Y-27632 partially restored the decreased BMAL1 expression induced by excessive loading. These results indicate that mechanical signals potentially regulate the peripheral clock via the RhoA/ROCK pathway. In addition, BMAL1 knockdown by siRNA led to decreased aggrecan expression and increased MMP13 expression, whereas Y-27632 did not rescue the si-Bmal1-induced degenerative phenotype. These results further demonstrate that RhoA/ROCK pathway activation by excessive loading potentially results in decreased BMAL1 expression, leading to ECM metabolism disruption (Fig. 7b). Cao Yang et al. recently reported that compression stress could induce RhoA/ROCK1 pathway activation, thus regulating the interaction of myosin IIA and IIB with actin.^23^ Our findings regarding compression-induced RhoA/ROCK activation were consistent with their conclusions, and the myosin-actin interaction may be a potential mechanism underlying decreased BMAL1 expression.

Clock-based therapeutic strategies have been proposed as promising therapeutic methods for many degenerative diseases, such as hypercholesterolemia, hypertension, endocrine disorders, and even cancers.^47–51^ However, an effective clock-based therapeutic strategy for IDD is still lacking. Our work innovatively demonstrated that RhoA/ROCK pathway inhibition could recover compression-induced decreased BMAL1 expression in NP cells, and two potential IDD chronotherapy drugs (Y-27632 and melatonin) were successfully used (Fig. 7b). Melatonin has been widely used for the treatment of many degenerative diseases, and multiple biological effects of melatonin, such as maintaining the central clock and antioxidation, antiaging, antiapoptotic, and anti-inflammatory effects, have been confirmed.^52–55^ Zheng et al. reported that melatonin could alleviate IDD by disrupting the IL-1β/NF-κB-NLRP3 inflammasome positive feedback loop^52^. Our results also confirmed the protective effects of melatonin on the process of IDD and further demonstrated that melatonin could ameliorate excessive loading-induced circadian disruption of NP cells by inhibiting the RhoA/ROCK pathway and thus attenuate the process of IDD. Considering the side effects of systemic administration, intradiscal injections were accepted.

An inevitable limitation of this study is that static compression stress in a rat model cannot fully imitate excessive mechanical loading in daily life in humans. To better mimic the abnormal mechanical loading caused by constant working and avoid the side effects of continuous static compression caused by traditional Ilizarov-type apparatuses, we invented a novel adjustable apparatus offering quantified IVD mechanical loading (Fig. 7c). The new apparatus was convenient for daily adjustment of the quantity of compression and reduced the injury induced by Kirschner wires. Additionally, although we have confirmed that inhibiting the RhoA/ROCK pathway is an effective method to recover the compression-induced decrease in BMAL1 expression, how RhoA/ROCK signaling regulates the clock protein BMAL1 has not been illustrated. Further studies to reveal the mechanism are still required.

In conclusion, excessive loading dampens the IVD circadian clock. Moreover, our results indicated that upregulating BMAL1 expression by RhoA/ROCK pathway inhibition attenuated compression-induced IDD (Fig. 7a–c). These results provide new insights into IDD development and offer a new therapeutic strategy for IDD prevention.

## Materials and methods

### Patient samples

One hundred forty-nine NP specimens were obtained from 98 patients (45 men, mean age = 50.62 ± 15.34 years; 53 women, mean age = 55.70 ± 11.12 years) with degenerative disc disease or scoliosis. All surgical samples were obtained during the first operation in the morning. None of the donors had neurologic abnormalities, endocrine system abnormalities, or autoimmune system abnormalities (insomnia, diabetes mellitus, hyperthyroidism, adrenal function hypoplasia, rheumatoid arthritis, systemic lupus erythematosus, ankylosing spondylitis, etc.) and signed informed consent before surgery. IDD severity was assessed by three blinded spine surgeons according to the modified Pfirrmann grading system by magnetic resonance imaging (MRI). Grade II (*n* = 14, mean age = 44.07 ± 15.16 years) and III (*n* = 52, mean age = 51.93 ± 11.61 years) samples were merged into a moderate group (mean age = 50.07 ± 12.85 years), and Grade IV (*n* = 59, mean age = 57.82 ± 10.80 years) and V (*n* = 24, mean age = 57.67 ± 12.95 years) samples were merged into a severe group (mean age = 57.77 ± 11.44 years). Specimen data are shown in Table S1. Ethical approval was obtained from the Institutional Review Board of Xijing Hospital of the Fourth Military Medical University (KY20203146-1). The study was conducted according to the Code of Ethics of the World Medical Association (Declaration of Helsinki).

### Animals

All animal experiments were approved by the Animal Use and Care Committee of the Fourth Military Medical University and conducted in accordance with the National Institute for Health Guide for the Care and Use of Laboratory Animals. PER2::Luc mice carry the firefly luciferase gene fused in frame with the *Per2* gene’s 3’ end, creating a fusion protein reporter^56^. *Bmal1*^*flox/flox*^ mice were crossed with *Col2a1*^*cre*^ mice to generate cartilage/IVD-specific *Bmal1* knockout mice. The genotyping method has been described before.^4,57^

### Reagents and antibodies

Forskolin and melatonin were purchased from Sigma-Aldrich. Y-27632 and arachidonic acid (AA) were purchased from Selleck Chemicals. The following antibodies were used in this study: BMAL1 (Abcam ab3350 for immunofluorescence and Santa Cruz Biotechnology sc-365645 for western blot), aggrecan (Millipore Sigma AB1031), MMP13 (Abcam ab39012 for western blot and Proteintech 18165-1-AP for immunofluorescence), and phosphomyosin light chain 2 (Ser19; Cell Signaling Technology 3671). Horseradish peroxidase (HRP)-conjugated β-actin mouse monoclonal antibody, HRP-conjugated AffiniPure goat anti-mouse or goat anti-rabbit IgG (H + L), fluorescein (FITC)-conjugated AffiniPure goat anti-rabbit IgG (H + L), and Cy3-conjugated AffiniPure goat anti-rabbit IgG (H + L) were purchased from Proteintech. Cell culture reagents were purchased from Gibco.

### Cell culture, tissue explant cultures, and bioluminescence recording

After enzymatic digestion (0.1% type II collagenase and 0.1% hyaluronidase) in serum-free Dulbecco’s modified Eagle’s medium (DMEM) overnight in a 5% CO_2_ incubator at 37 °C, human NP cells were cultured in DMEM supplemented with 10% FBS, 1 mmol·L^−1^ sodium pyruvate, 1% antibiotics (penicillin/streptomycin), 25 μg·mL^−1^ amphotericin B, and 1 mmol·L^−1^ ascorbate under standard conditions. Grade 2 cells were used for *Per2::luc* lentiviral transduction and bioluminescence photon counting.^58^ The culture of NP cells was described in a previous study.^59^ Rat NP cells were used at passages 5–8, and human NP cells were used at passage 2. The method for primary human NP cell lentiviral transduction and synchronization has been described previously.^30^

Organotypic IVD tissue explants were cultured as previously described.^30,60,61^ PER2::Luc mouse explants were cultured in 0.4-μm cell culture inserts (Millipore), and bioluminescence was recorded using a LumiCycle apparatus (ActiMetrics). Statistical and computational methods were performed as previously reported. Rat explants were cultured in 12-well culture plates. Compression stress was exerted by a pressure incubator according to our previous studies.^62–64^ The culture system comprised a compression culture chamber, electrothermostatic water bath, and controllable gas cylinder (filled with 76% N_2_, 19% O_2_, and 5% CO_2_). Rat explants were subjected to 0 MPa (resting condition) or 1.0 MPa (excessive mechanical loading) for 6 and 12 days (complete medium was changed every other day), and NP cells were subjected to 0 MPa or 1.0 MPa for 0, 12, 24, 36, and 48 h, and we selected 24 h for most experiments.

### Western blot assay

The western blot assay method has been previously described.^59^ NP cells were lysed in RIPA buffer containing protease and phosphatase inhibitors (Beyotime). After centrifugation (4 °C, 12 000 r·min^−1^, 10 min), the supernatant was collected, and the total protein concentration was determined using the BCA Protein Assay Kit (Beyotime). Each sample (30 mg total protein) was separated in sodium dodecyl sulfate-polyacrylamide gel (Beyotime) and transferred to nitrocellulose filter membranes (Millipore). After blocking with western blocking buffer (Beyotime) for 1 h at 37 °C, the membranes were incubated with primary anti-BMAL1 (1:1 000), aggrecan (1:1 000), MMP13 (1:1 500), pMLC (1:1 000), and β-actin antibodies (1:2 000) at 4 °C overnight. The membranes were then incubated with the appropriate HRP-conjugated secondary antibodies (1:2 000) for 1 h at 37 °C. Finally, the bands were detected using ECL-Plus Reagent (Millipore) and observed under an Amersham Imager 600 (General Electric).

### Immunofluorescence staining

NP cells on the slides were initially fixed in 4% paraformaldehyde (Beyotime) for 15 min. The cell slides or histological sections were permeabilized using 0.3% Triton X-100 (Beyotime) for 10 min and immersed in QuickBlock™ Blocking Buffer for Immunol Staining (Beyotime) for 1 h. After incubation with primary anti-BMAL1 (1:200), MMP13 (1:50), and aggrecan antibodies (1:100) at 4 °C overnight, cell slides or histological sections were immersed in appropriate FITC- or Cy3-conjugated secondary antibodies (1:200) at 37 °C for 2 h and subsequently incubated with 4’,6-diamidino-2-phenylindole (Beyotime) for 5 min. Each step was followed by washing with PBS three times for 5 min. The cells or sections were observed under a fluorescence microscope, and fluorescence intensity was quantified using ImageJ software.

### Apoptosis assay

An apoptosis assay was performed on rat IVD sections and NP cell slides by terminal deoxynucleotidyl transferase dUTP nick end labeling (TUNEL) staining using the One-Step TUNEL Apoptosis Assay Kit (Beyotime) and flow cytometry with Annexin V-FITC/PI (BD Biosciences). These experiments were conducted as previously reported,^59^ and TUNEL-positive cells were calculated using ImageJ software.

### Surgical procedure and histopathological analysis

Six male 5-month-old Sprague–Dawley rats were prepared for the research. All rats were anesthetized with isoflurane, and a three-level IDD model was established at the Co7–8, Co8–9, and Co9–10 levels. To facilitate daily loading and drug injection into the IVD, we developed a novel adjustable mechanical loading apparatus based on a traditional Ilizarov-type apparatus.^65–68^ We imposed excessive mechanical loading on the rat IVDs at 4:00 pm and removed the compression at 8:00 am. Three IVDs (Co7–8, Co8–9, and Co9–10) were randomly selected for intradiscal injection of phosphate-buffered saline (PBS), melatonin (500 μmol·L^−1^), and Y-27632 (10 μmol·L^−1^). One of the two IVDs (Co7–8 and Co10–11) was randomly accepted for intradiscal PBS injection as the control group. Intradiscal treatment was performed semiweekly. All animal experiments in this study followed the International Guiding Principles for Biomedical Research Involving Animals.

The rats were sacrificed 28 days after surgery by inhaling an excessive amount of isoflurane, and the spine specimens were subsequently harvested. The specimens were fixed in 4% paraformaldehyde for 2 days, decalcified in 10% ethylenediaminetetraacetic acid (pH 7.4) for 45 days, and embedded in paraffin. Midsagittal-oriented sections (5 mm) were prepared for hematoxylin–eosin (HE) and safranin O-fast green (SO) staining according to the respective staining kit protocols (Solarbio, Beijing, China). Histological scores were evaluated in a blinded manner according to a previously described protocol.^69^

### X-ray, micro-CT, and MRI assay

Twenty-eight days after the initial surgical procedure, six rats were randomly selected for radiography and MRI before they were sacrificed. Lateral radiographs were taken at each time point (exposure time, 0.06 s; distance, 100 cm; current, 160 mA; voltage, 50 kV) using the DRX-Ascend system (Carestream). Prone-position MRI was performed using a 3.0 T system (GE) to obtain T2-weighted images (repetition time, 1 600 ms; echo time, 85 ms; field of view, 80 × 80 mm; slice thickness, 2.0 mm). Micro-CT was performed on 6-month-old mice (four wild-type [WT] and four *Col2a1*^*Cre*^*Bmal1*^*fl/fl*^ [cKO]) for quantitative disc height evaluation. The NP water content was detected by MRI T2-weighted images (repetition time, 1 800 ms; echo time, 102 ms; field of view, 49 × 49 mm; slice thickness, 1.5 mm). The relative signal intensity for mouse NP tissues was calculated using ImageJ software.

### Statistical analysis

Data are expressed as the mean ± standard error of the mean. Single-factor linear regression was used to analyze the relationship between the BMAL1 positivity rate and Pfirrmann grade (or age). Multivariate linear and logistic regression analyses were used to model the relationship between all variables (BMAL1 positivity rate, sex, and age) and Pfirrmann grades. Rat Pfirrmann grades and histological scores among multiple groups were analyzed using the Kruskal–Wallis h-test. Between-group differences were analyzed using Student’s t test or one-way ANOVA. All statistical analyses were performed using GraphPad Prism software (version 7.0) and SPSS 22.0. Differences were considered significant at *P < 0.05 and **P < 0.01.

## Supplementary information

supplementary materials

## Data Availability

The raw data supporting the conclusions of this article will be made available by the authors, without undue reservation.
