# Supplementary material for: Restoring the dampened expression of the core clock molecule BMAL1 protects against compression-induced intervertebral disc degeneration
Source: Bone Res. 2022 Feb 25;10:20. doi: 10.1038/s41413-022-00187-z (PMC8881495; doi:10.1038/s41413-022-00187-z)

**Supplementary table S1: Demographic data of patients**

| Patient no | Age | Gender | Levels and Pfirrmann grading |
| --- | --- | --- | --- |
| 1 | M | 55 | L2-L3: Ⅲ |
| 2 | M | 29 | L5-S1: Ⅴ |
| 3 | M | 37 | L4-L5: Ⅲ; L5-S1: Ⅴ |
| 4 | M | 55 | L3-L4: Ⅱ; L4-L5: Ⅲ |
| 5 | M | 72 | L3-L5: Ⅳ; L5-S1: Ⅴ |
| 6 | M | 37 | L5-S1: Ⅲ |
| 7 | M | 68 | L3-L4: Ⅳ; L4-L5: Ⅳ |
| 8 | M | 57 | L5-S1: Ⅲ |
| 9 | M | 45 | L3-L4: Ⅱ; L4-L5: Ⅲ |
| 10 | M | 65 | L4-L5: Ⅳ; L5-S1: Ⅳ |
| 11 | M | 56 | L3-L5: Ⅳ; L5-S1: Ⅴ |
| 12 | M | 54 | L5-S1: Ⅴ |
| 13 | M | 57 | L3-L4: Ⅲ; L4-L5: Ⅴ |
| 14 | M | 76 | L3-L4: Ⅳ |
| 15 | M | 22 | L4-L5: Ⅱ |
| 16 | M | 34 | L5-S1: Ⅳ |
| 17 | M | 55 | L2-L3: Ⅳ; L3-L4: Ⅳ; L4-L5: Ⅳ |
| 18 | M | 47 | L2-L3: Ⅲ |
| 19 | M | 30 | L5-S1: Ⅱ |
| 20 | M | 61 | L3-L4: Ⅲ; L4-L5: Ⅲ |
| 21 | M | 29 | L5-S1: Ⅲ |
| 22 | M | 65 | L4-L5: Ⅳ |
| 23 | M | 39 | L5-S1: Ⅲ |
| 24 | M | 71 | L3-L4: Ⅲ; L4-L5: Ⅲ |
| 25 | M | 49 | L5-S1: Ⅳ |
| 26 | M | 30 | L4-L5: Ⅳ |
| 27 | M | 40 | L5-S1: Ⅱ |
| 28 | M | 58 | L2-L3: Ⅱ |
| 29 | M | 44 | L5-S1: Ⅲ |
| 30 | M | 39 | L5-S1: Ⅳ |
| 31 | M | 32 | L4-L5: Ⅲ |
| 32 | M | 74 | L3-L4: Ⅳ; L4-L5: Ⅳ |
| 33 | M | 52 | L2-L3: Ⅳ; L3-L4: Ⅴ; L4-L5: Ⅴ |
| 34 | M | 62 | L5-S1: Ⅳ |
| 35 | M | 56 | L5-S1: Ⅲ |
| 36 | M | 40 | L5-S1: Ⅲ |
| 37 | M | 71 | L1-L2: Ⅴ |
| 38 | M | 49 | L4-L5: Ⅲ |
| 39 | M | 35 | L4-L5: Ⅱ |
| 40 | M | 66 | L4-L5: Ⅲ; L5-S1: Ⅳ |
| 41 | M | 33 | L4-L5: Ⅲ |
| 42 | M | 68 | L3-L4: Ⅲ; L4-L5: Ⅳ |
| 43 | M | 71 | L3-L4: Ⅳ; L4-L5: Ⅲ |
| 44 | M | 25 | T11-T12: Ⅱ |
| 45 | M | 68 | L5-S1: Ⅲ |
| 46 | F | 55 | L3-L4: Ⅴ; L4-S1: Ⅲ |
| 47 | F | 43 | L3-L4: Ⅳ; L4-L5: Ⅳ; L5-S1: Ⅴ |
| 48 | F | 53 | L4-L5: Ⅲ; L5-S1: Ⅱ |
| 49 | F | 70 | L4-L5: Ⅳ; L5-S1: Ⅴ |
| 50 | F | 64 | L4-L5: Ⅴ |
| 51 | F | 64 | L5-S1: Ⅴ |
| 52 | F | 44 | L4-L5: Ⅴ; L5-S1: Ⅲ |
| 53 | F | 58 | L4-L5: Ⅳ |
| 54 | F | 27 | L4-S1: Ⅲ |
| 55 | F | 51 | L3-L4: Ⅲ; L4-L5: Ⅳ |
| 56 | F | 47 | L5-S1: Ⅳ |
| 57 | F | 64 | L5-S1: Ⅴ |
| 58 | F | 64 | L3-L4: Ⅳ; L4-L5: Ⅳ |
| 59 | F | 29 | L5-S1: Ⅱ |
| 60 | F | 51 | L3-L4: Ⅲ; L4-L5: Ⅳ |
| 61 | F | 46 | L5-S1: Ⅲ |
| 62 | F | 56 | L2-L3: Ⅲ; L4-L5: Ⅳ |
| 63 | F | 42 | L5-S1: Ⅴ |
| 64 | F | 49 | L3-L4: Ⅳ; L4-L5: Ⅳ |
| 65 | F | 53 | L3-L4: Ⅳ |
| 66 | F | 77 | L4-L5: Ⅴ |
| 67 | F | 50 | L4-L5: Ⅳ |
| 68 | F | 66 | L4-L5: Ⅳ; L5-S1: Ⅳ |
| 69 | F | 62 | L4-L5: Ⅳ; L5-S1: Ⅳ |
| 70 | F | 56 | L4-L5: Ⅲ; L5-S1: Ⅱ |
| 71 | F | 56 | L4-L5: Ⅳ; L5-S1: Ⅳ |
| 72 | F | 64 | L3-L4: Ⅲ; L4-L5: Ⅳ |
| 73 | F | 53 | L4-L5: Ⅲ; L5-S1: Ⅲ |
| 74 | F | 72 | L5-S1: Ⅴ |
| 75 | F | 65 | L3-L4: Ⅴ; L4-L5: Ⅴ |
| 76 | F | 52 | L4-L5: Ⅳ |
| 77 | F | 54 | L4-L5: Ⅳ; L5-S1: Ⅳ |
| 78 | F | 50 | L3-L4: Ⅲ; L4-L5: Ⅲ; L5-S1: Ⅲ |
| 79 | F | 36 | L5-S1: Ⅱ |
| 80 | F | 68 | L4-L5: Ⅴ |
| 81 | F | 60 | L3-L4: Ⅲ; L4-L5: Ⅲ |
| 82 | F | 35 | L5-S1: Ⅲ |
| 83 | F | 43 | L5-S1: Ⅲ |
| 84 | F | 67 | L4-L5: Ⅳ; L5-S1: Ⅳ |
| 85 | F | 63 | L3-L4: Ⅳ; L4-L5: Ⅲ |
| 86 | F | 75 | L3-L4: Ⅳ; L4-L5: Ⅳ |
| 87 | F | 65 | L3-L4: Ⅱ; L4-L5: Ⅲ |
| 88 | F | 54 | L4-L5: Ⅳ |
| 89 | F | 68 | L4-L5: Ⅱ; L5-L1: Ⅲ |
| 90 | F | 63 | L5-S1: Ⅲ |
| 91 | F | 51 | L3-L4: Ⅲ; L4-L5: Ⅲ; L5-S1: Ⅳ |
| 92 | F | 51 | L4-L5: Ⅳ |
| 93 | F | 62 | L4-L5: Ⅲ |
| 94 | F | 49 | L4-L5: Ⅲ |
| 95 | F | 54 | L4-L5: Ⅳ |
| 96 | F | 51 | L4-L5: Ⅳ |
| 97 | F | 75 | L3-L4: Ⅳ; L4-L5: Ⅳ |
| 98 | F | 55 | L1-L2: Ⅲ: L2-L3: Ⅳ; L3-L4: Ⅴ; L4-L5: Ⅴ |

F=female; M=male.

**Supplementary table S2: Logistic regression analysis of the participants in the process of IDD**

|  | B | SE | Wald | df | Significant | Exp(B) | 95% Cl for Exp(B) | |
| --- | --- | --- | --- | --- | --- | --- | --- | --- |
|  |  |  |  |  |  |  | Lower | Upper |
| BMAL1 Positive Rate | -0.096 | 0.016 | 38.233 | 1 | 0.000 | 0.908 | 0.881 | 0.936 |
| Ages | 0.011 | 0.022 | 0.243 | 1 | 0.622 | 1.011 | 0.968 | 1.057 |
| Gender | -0.571 | 0.491 | 1.352 | 1 | 0.245 | 0.565 | 0.216 | 1.479 |
| Constant | 4.725 | 1.688 | 7.837 | 1 | 0.005 | 112.777 |  |  |

**Supplementary figures S1.**

a. Quantification of the fluorescence intensity of Aggrecan, BMAL1 and MMP13 in Fig.3a.

b. Quantification of the fluorescence intensity of Aggrecan in Fig.3f.


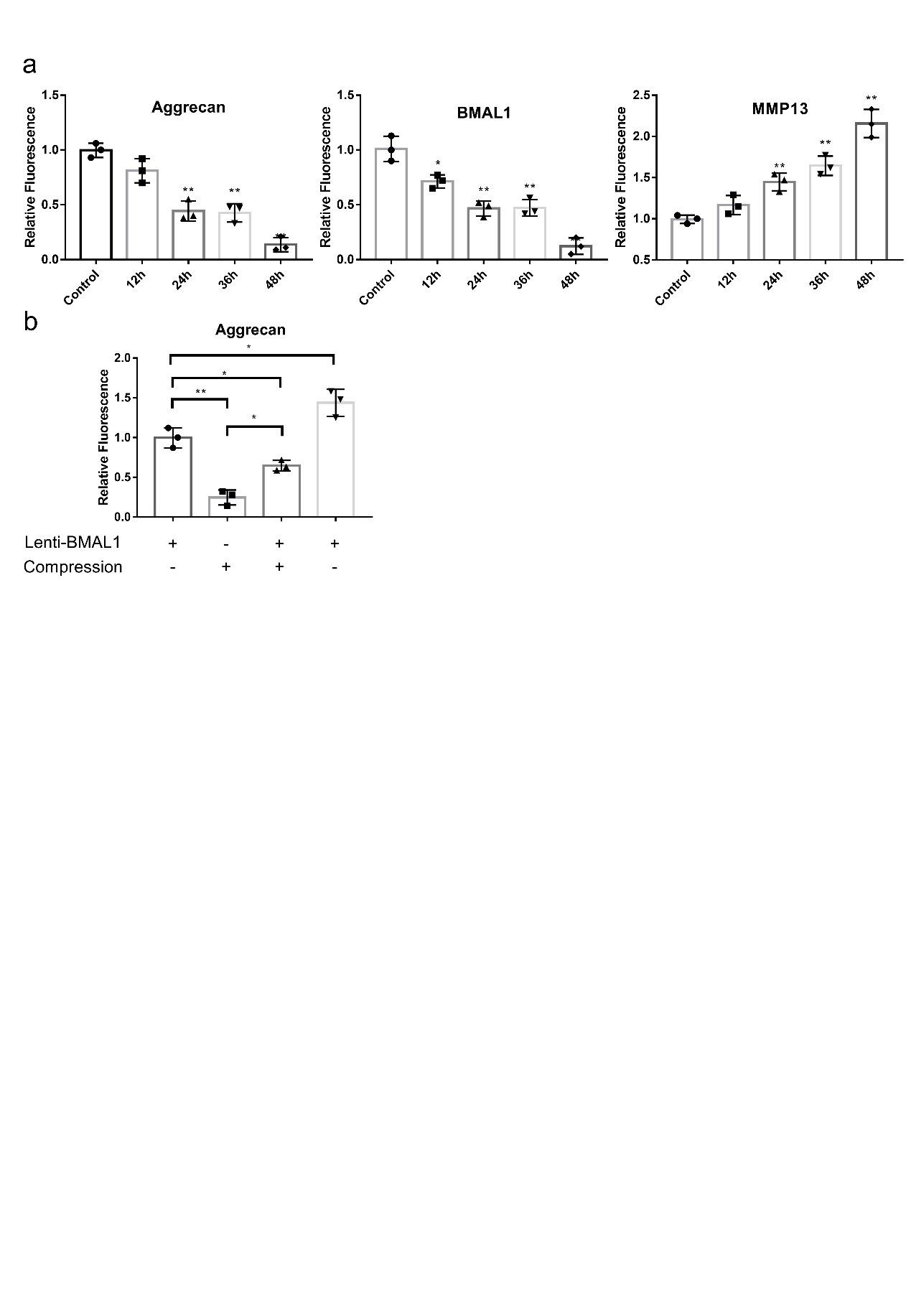


**Supplementary figures S2. Inhibiting RhoA/ROCK pathway ameliorated compression induced the apoptosis of NPCs and degradation of ECM in NP tissues. a** Representative HE, SO, and TUNEL staining images of organotypic tissue-explants on day 6. **b** Representative HE, SO, and TUNEL staining images of organotypic tissue-explants on day 12.


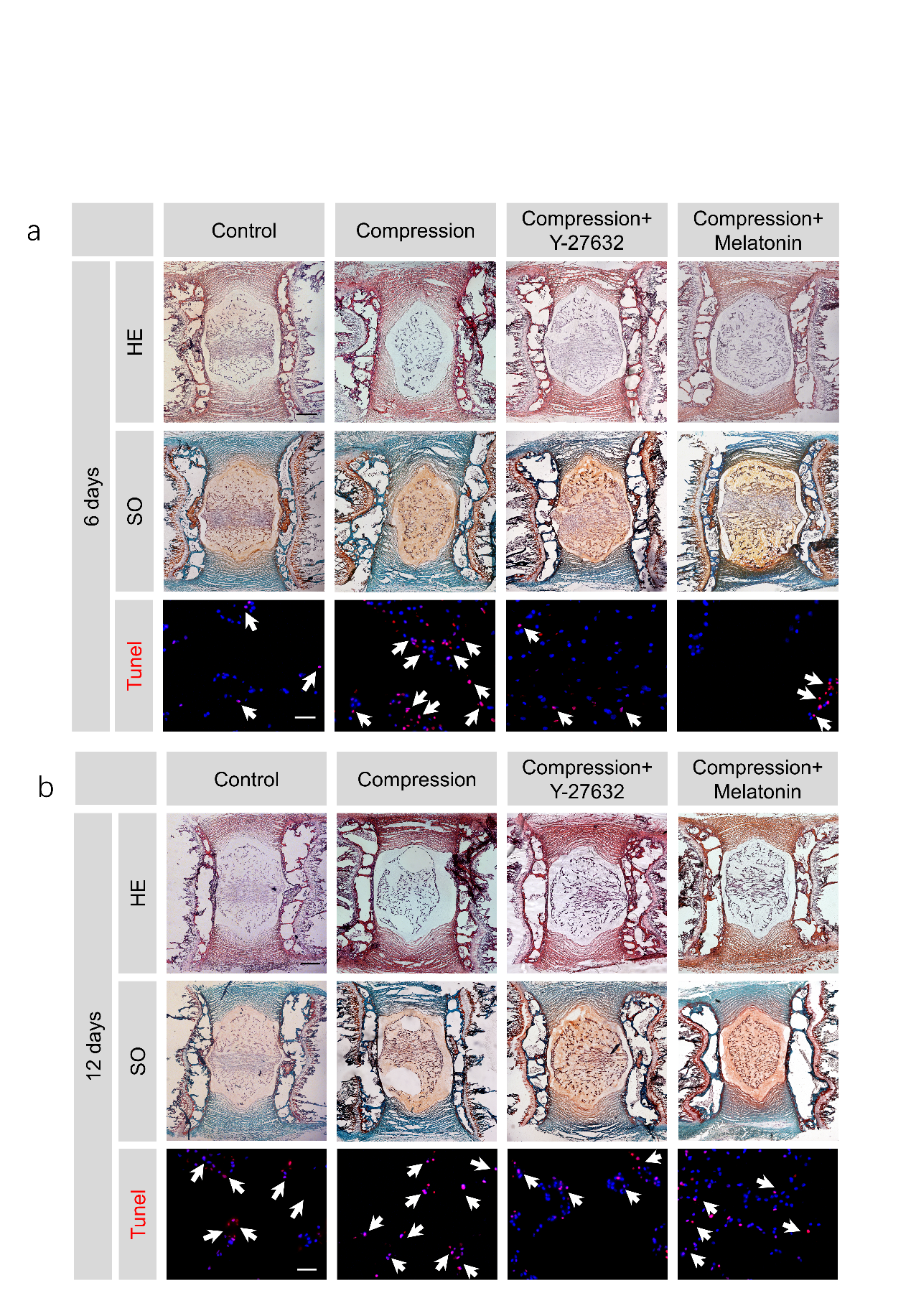


**Supplementary figures S3.**

a. Quantification of the fluorescence intensity of Aggrecan, BMAL1 and MMP13 in Fig.6e.


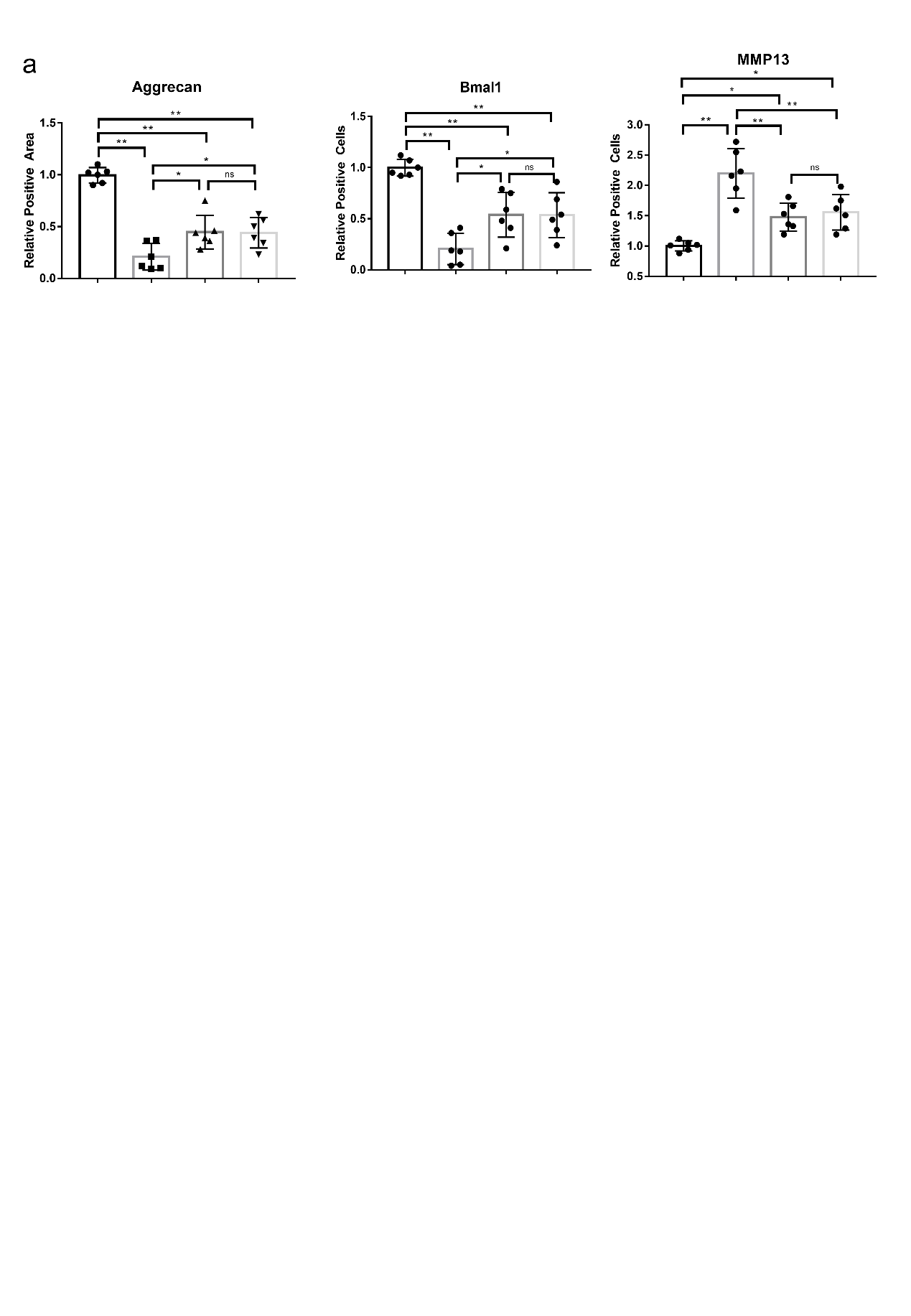

Supplement: Supplementary file 1 — supplementary materials [file 41413_2022_187_MOESM1_ESM.docx]
